# Regulation of EMT Markers, Extracellular Matrix, and Associated Signalling Pathways by Long Non-Coding RNAs in Glioblastoma Mesenchymal Transition: A Scoping Review

**DOI:** 10.3390/biology12060818

**Published:** 2023-06-04

**Authors:** Dexter Hoi Long Leung, Brandon Wee Siang Phon, Mageswary Sivalingam, Ammu Kutty Radhakrishnan, Muhamad Noor Alfarizal Kamarudin

**Affiliations:** Jeffrey Cheah School of Medicine and Health Sciences, Monash University Malaysia, Jalan Lagoon Selatan, Bandar Sunway 47500, Malaysia

**Keywords:** glioblastoma, mesenchymal transition markers, long non-coding RNA, extracellular matrix, EMT markers

## Abstract

**Simple Summary:**

Glioblastoma (GBM) is the most lethal type of brain tumour due to the high invasiveness caused by the process of mesenchymal (MES) transition. This process is modulated by a myriad of biological factors, one of them being long non-coding RNAs (lncRNAs) which remain highly elusive in the GBM. In this scoping review, all recent lncRNAs which were studied to play a regulatory role in GBM cells through modulating EMT markers, transcription factors (TFs), and the affiliated signalling pathways associated with MES transition were identified through a systematic literature search and reviewed. The results provide an update and prospective on the complex interplays of the EMT markers, TFs, and signalling pathways with lncRNAs identified in GBM cell studies.

**Abstract:**

Glioblastoma (GBM) mesenchymal (MES) transition can be regulated by long non-coding RNAs (lncRNAs) via modulation of various factors (Epithelial-to-Mesenchymal (EMT) markers, biological signalling, and the extracellular matrix (ECM)). However, understanding of these mechanisms in terms of lncRNAs is largely sparse. This review systematically analysed the mechanisms by which lncRNAs influence MES transition in GBM from a systematic search of the literature (using PRISMA) performed in five databases (PubMed, MEDLINE, EMBASE, Scopus, and Web of Science). We identified a total of 62 lncRNAs affiliated with GBM MES transition, of which 52 were upregulated and 10 were downregulated in GBM cells, where 55 lncRNAs were identified to regulate classical EMT markers in GBM (E-cadherin, N-cadherin, and vimentin) and 25 lncRNAs were reported to regulate EMT transcription factors (ZEB1, Snai1, Slug, Twist, and Notch); a total of 16 lncRNAs were found to regulate the associated signalling pathways (Wnt/β-catenin, PI3k/Akt/mTOR, TGFβ, and NF-κB) and 14 lncRNAs were reported to regulate ECM components (MMP2/9, fibronectin, CD44, and integrin-β1). A total of 25 lncRNAs were found dysregulated in clinical samples (TCGA vs. GTEx), of which 17 were upregulated and 8 were downregulated. Gene set enrichment analysis predicted the functions of HOXAS3, H19, HOTTIP, MEG3, DGCR5, and XIST at the transcriptional and translational levels based on their interacting target proteins. Our analysis observed that the MES transition is regulated by complex interplays between the signalling pathways and EMT factors. Nevertheless, further empirical studies are required to elucidate the complexity in this process between these EMT factors and the signalling involved in the GBM MES transition.

## 1. Introduction

IDH-wildtype Glioblastoma (GBM) denotes the most aggressive form of adult-type diffuse glioma with a low survival and high rate of tumour recurrence post-surgical resection [1]. This phenomenon is greatly attributed to the high invasiveness of the glioblastoma (GBM), which makes the complete removal of this tumour difficult, leading to residual tumour foci that can grow and develop resistance to therapy [2]. GBM can also be classified into neural, pro-neural, classical, or mesenchymal subtypes based on differences in genetic signatures, whereby the mesenchymal (MES) subtype is associated with the highest invasive capability [3]. In a healthy brain environment, the dysregulation of various interplays amongst the biological processes at the genetic level may lead to the development of aggressive phenotypes, often seen in malignant tumours [4]. The highly invasive characteristics of the MES subtype of GBM are attributed to a biological event which induces GBM cells of other subtypes to transition to the MES state—an event which is termed as MES transition. MES transition is highly similar to the classic EMT process observed in the epithelial setting and is a major driver that allows GBM cells to exhibit an aggressive phenotype [5].

In the epithelial setting, EMT is a process where the epithelial cells transition into the mesenchymal state, which facilitates cell migration by allowing these cells to detach from a basal membrane [6]. It is a dynamic process that involves disruption of cell–cell adhesion, cytoskeleton remodelling, and changes in the cell–matrix behaviour, all of which ultimately affect tumour progression and metastasis [7]. This process is tightly regulated and is often observed in various cancers. The commonly used markers for the classical EMT process are commonly recognised as E-cadherin, N-cadherin, and vimentin. Induction of the EMT process is characterised by the downregulation of E-cadherin and the upregulation of N-cadherin and vimentin, which are responsible for the loss of cell-to-cell adhesion [8]. The expression of cadherins is governed by several transcription factors; namely ZEB1/2, Snail transcription family (Snail/Slug), Twist1, and Notch [9]. In addition, these transcription factors can also modulate the activation of specific signalling pathways such as mTOR, PI3K/Akt, Wnt/β-catenin, and TGF-β pathways, which affect the EMT process [10]. Because GBMs are not typical epithelial cells due to the absence of a basal membrane within the neural setting and inconsistent expression of E-cadherin, to coin the term “EMT” within the GBM context would be scientifically inaccurate. However, studies in recent years have found that the regulation of the classical EMT markers can induce the GBM cells to the invasive mesenchymal (MES) subtype [11]. These observations have recorded the similarities in genetic profiles observed in both MES transition and the classical EMT process, which includes the classical EMT markers, TFs, and induction of signalling pathways. In the neural setting, the highly hypoxic microenvironment and the extracellular matrix (ECM) are factors which affect GBM MES transition [12]. The highly hypoxic tumour microenvironment stimulates hypoxic adaptive responses, which in turn induces MES transition. Tumour cells will then become increasingly motile where they traverse the ECM which involves cellular interactions with both matrix and cytoskeletal proteins. This will lead to the changes in the integrity of the ECM, leading to cytoskeletal remodelling which further supports the pro-invasion activities of GBM cells.

Looking at the research trend within the past few years, non-coding RNAs (ncRNAs) are known to regulate a wide range of biological processes and signalling pathways, which include cancers and their associated migratory and metastasis mechanisms that include the EMT process. The ncRNAs have garnered interest in cancer research due to their ability to act as molecular “switches” of various signalling pathways in regulating cellular activities through a competitive endogenous RNA (ceRNA) mechanism. The ncRNAs will “sponge” their target through this mechanism, resulting in a silencing effect at both the translational and protein levels [13]. Long non-coding RNAs (lncRNAs) are a class of ncRNAs with more than 200 nucleotides that can sponge with the target microRNAs (miRNAs), DNA, and proteins, which subsequently affect downstream processes. With the growing number of new lncRNAs, their characterisation is essential to complement the current knowledge enshrouding their roles in malignant tumour progression. In cancers, a huge number of lncRNAs and their the involvements in both cancers and EMT are well characterised [14]. Various pro- and anti-EMT lncRNAs have been deliberated in cancers which exhibited differential effects, whereby the regulation of EMT by lncRNA(s) was observed to be present at both the transcriptional and translational levels. The physiological role of lncRNAs is also context-dependent, where a single lncRNA can exhibit differential effects between different cancer subtypes. This multifaceted nature of lncRNAs has posed a challenge to effectively identify and characterise their molecular role in specific cancers. Although the development of lncRNA(s) is still at a very premature stage, they could be elicited as a promising target of prognostic or therapeutical value.

A recent systematic review on colorectal cancer identified several lncRNAs that regulate EMT via the ZEB1, E-cadherin, and Wnt/β-catenin pathways [15]. Although lncRNAs have been widely reported in EMT-related signalling pathways in GBM [16], the specific molecular mechanisms in the GBM MES transition are not fully elucidated. Hence, through this systematic scoping review, we aim to review and analyse the roles of various lncRNAs in regulating common EMT markers in GBM, transcription factors, signalling pathways, and ECM components, which may provide insights into GBM cell invasion via the involvement of the GBM MES transition. This analysis also further identifies their expression and relevancy in clinical samples and further bridges the gaps in understanding GBM MES transition regulation by clinically relevant lncRNAs in GBM.

## 2. Materials and Methods

### 2.1. Literature Search Strategy

The Preferred Reporting Items for Systematic Reviews and Meta-analyses (PRISMA) guidelines were used to design, analyse, and report data obtained in this scoping review. Five electronic databases, i.e., PubMed (NLM, Bethesda, MD, USA), MEDLINE (NLM, Bethesda, MD, USA), EMBASE (Elsevier, Amsterdam, The Netherlands), Scopus (Elsevier, Amsterdam, The Netherlands), and Web of Science (Clarivate Analytics, London, UK), were searched systematically for studies related to lncRNAs and their roles in GBM MES transition.

### 2.2. Study Selection

The search was restricted from 1 January 2011 to 31 October 2021. The following search terms were used: “Glioblastoma”, “Glioma”, “GBM”, “EMT”, “Epithelial-to Mesenchymal”, “Mesenchymal”, “Progression”, “ZEB”, “TWIST”, “SLUG”, “SNAI1”, “lncRNA”, “non-coding RNA”, “long non-coding RNA”. The Boolean search terms “AND” and “OR” were used to define the search. Searches from all databases were collected and duplicate references were removed manually. Only English original articles were included in the final selection for data analysis.

### 2.3. Inclusion and Exclusion Criteria

Articles were included in the study (i.e., met the inclusion criteria) if the article reported on original research on cell-based models of GBM associated with GBM MES transition or if they were studies investigating the molecular signalling role or involvements of lncRNAs in any type of cellular processes/molecular pathways or factors that will lead to GBM MES transition programme in GBM. In contrast, articles that did not focus on lncRNAs in GBM cell lines, involved in vivo studies, were unrelated to EMT-related mechanisms, or were review articles were excluded from the study (i.e., exclusion criteria).

### 2.4. Data Extraction and Outcomes

Title and abstract screening, full-text screening, and data extraction were performed by two researchers (DHLL and BWSP). All screenings required consensus between both researchers, and any conflicts were resolved by a senior author (MNAK). The following data were extracted from the selected studies: authors, year of publication, lncRNA, miRNA interaction, further signalling interaction(s), cell lines used, and relevance to GBM MES transition.

### 2.5. Validation of lncRNAs via TCGA, and GTEx Datasets

For validation, level 3 gene expression profiles of TCGA patient cohort and normal brain samples were obtained from UCSC Xena data portal (https://xenabrowser.net/datapages/, accessed on 20 November 2021) [17]. Specifically, Illumina Hiseq2000 RNASeq log_2_ transformed HTSeq counts were obtained. Clinical data of the GBM patients from the TCGA database such as gender, age, IDH1 mutation status, and sample type were obtained both from UCSC Xena [17] and cBioPortal (https://www.cbioportal.org, accessed on 20 November 2021) [18,19]. The utilisation of the Toil pipeline allowed for a unified processing workflow between the TCGA and GTEx datasets, with STAR being used to generate alignments and quantifications being performed using RSEM [20]. The recomputation of the raw RNA-Seq data from TCGA and GTEx by the UCSC Xena project makes the two datasets compatible, allowing for the direct expression analyses.

### 2.6. Gene Set Enrichment Analysis of lncRNAs

Protein interactions associated with the lncRNAs identified in our literature search and available in the TCGA vs. GTEx database were obtained from the RAID v2 database (https://www.rna-society.org/raid2/, accessed on 20 November 2021). Gene set enrichment analysis (GSEA) was then performed through Enrich (https://maayanlab.cloud/Enrichr/, accessed on 20 November 2021), where the Kyoto Encyclopedia of Genes and Genomes (KEGG) pathways, Gene Ontology (GO) Biological Process (BP), and GO Molecular Functions (MF) annotations were utilised.

## 3. Results

### 3.1. Literature Search Results of lncRNAs Regulating GBM MES Transition

**Figure 1:**
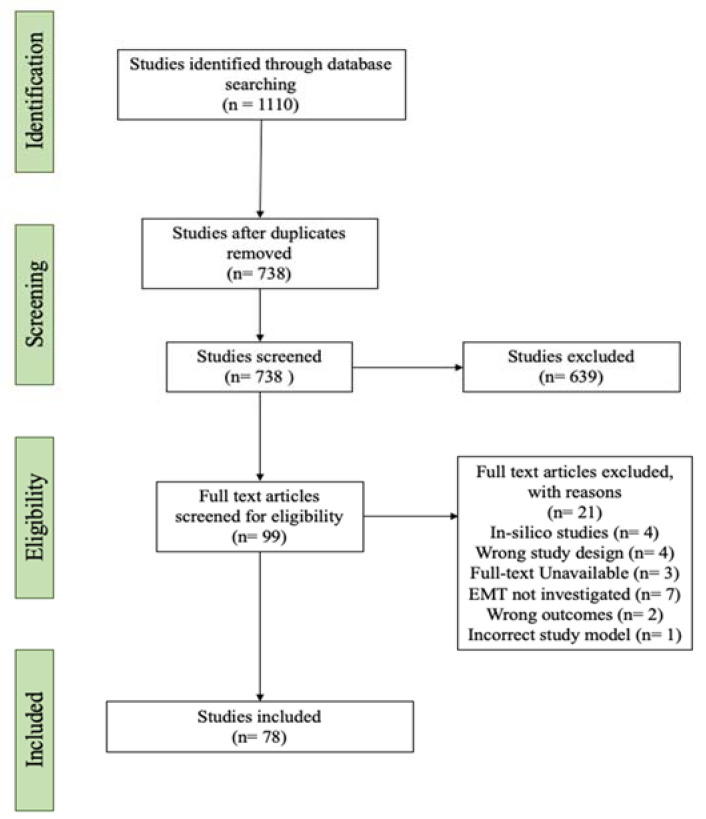
PRISMA flow diagram for systematic search of articles from PubMed, MEDLINE, EMBASE, Scopus, and Web of Science literature databases. Articles were then further scrutinised for suitability to be included in the study based on the inclusion and exclusion criteria. Finally, the included studies were extracted and analysed in this study.

A total of 1110 original articles were identified from the databases. The search process is illustrated in Figure 1. Of the 1110 papers identified, 738 studies were included after removal of duplicate studies. Then, 639 studies were removed following the initial assessment based on title and abstract screening, which yielded 99 studies which were subjected to full-text review. During full-text review, 21 studies were excluded, leaving 78 studies which were included for data syntheses (Figure 1). In these 78 studies, 52 lncRNAs were reported to be upregulated, and 10 lncRNAs were reported to be downregulated (Table 1). The expression of these lncRNAs was validated (by the respective authors) using (i) quantitative PCR (qPCR), where the authors compared the expression of lncRNA(s) between GBM cell lines or and/or GBM tissues against normal brain tissues/cells, or (ii) through bioinformatics studies using The Cancer Genome Atlas (TCGA) and GTEx databases. Based on these results, the expression of lncRNAs that are oncogenic and promote GBM MES transition were often upregulated and, in contrast, the lncRNAs associated with a tumour suppressive role were downregulated.

### 3.2. Dysregulated lncRNAs Affect the Expression of Classical EMT Markers

**Figure 2:**
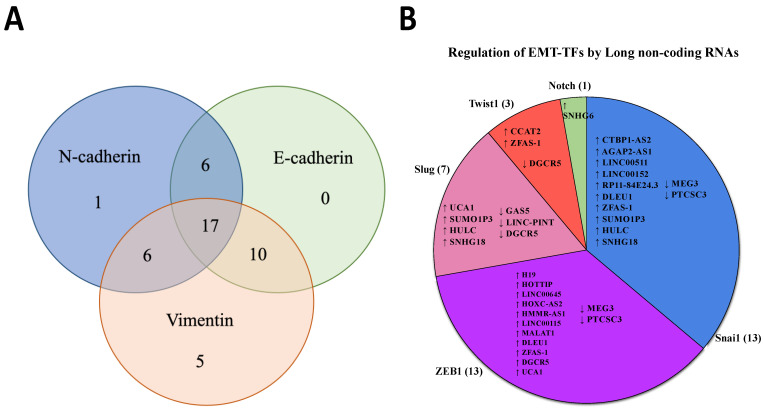
(**A**) The number of upregulated lncRNAs identified to regulate N-cadherin, E-cadherin, and vimentin expression. (**B**) The number of upregulated (↑) and downregulated (↓) lncRNAs identified through the systematic literature search which regulate EMT-TFs; Snai1, ZEB1, Slug, Twist1, and Notch.

LncRNAs can modulate GBM MES transition through direct or indirect regulation of the expression of common MES-related markers (vimentin, E-cadherin, and N-cadherin) through specific biological pathways. In most studies, the EMT markers commonly used to determine the MES transition are E-cadherin, N-cadherin, and vimentin. In our analysis, 45 upregulated and 10 downregulated lncRNAs can regulate the GBM MES transition through the direct change of expression of these EMT markers (Figure 2A, Table 1). A total of 30 lncRNAs were found to affect the expression of N-cadherin, and 33 and 38 lncRNAs regulated the expression of E-cadherin and vimentin, respectively (Table 1). Seventeen lncRNAs were identified to affect the expression of all three markers (E-cadherin, N-cadherin, and vimentin), while six lncRNAs (LINC00473, AGAP2-AS1, SAMMSON, NNT-AS1, ZFAS-1, and ZEB1-AS1) were found to affect the expression of both N-cadherin and E-cadherin; six lncRNAs (PDIA3P1, LINC00466, PVT1, HSP90AA1-IT1, HOXC13-AS, and SNHG11) were found to affect the expression of both N-cadherin and vimentin; and finally, ten lncRNAs were identified to affect the expression of both vimentin and E-cadherin (Table 1). In all studies, the regulation of E-cadherin is inversely proportional to the regulation of both N-cadherin and vimentin. Still, the regulation of both N-cadherin and vimentin appears directly proportional, signifying their roles as MES markers.

Based on the analysis, the upregulation of the oncogenic lncRNAs demonstrated a positive correlation with the increased expression of N-cadherin and vimentin, with a negative expression of E-cadherin. This observation was consistent with the knockdown of the upregulated lncRNAs via RNAi, demonstrating an increased expression of E-cadherin and a reduction in both mesenchymal markers. Meanwhile, overexpression of tumour suppressive lncRNAs led to the increased expression of E-cadherin and decreased N-cadherin and vimentin. These observations further support the oncogenic or tumour-suppressive roles of these lncRNAs as either a promoter or inhibitor of MES transition in GBM.

### 3.3. The Expression of Long Non-Coding RNAs Regulates EMT Transcriptional Factors

**Figure 3:**
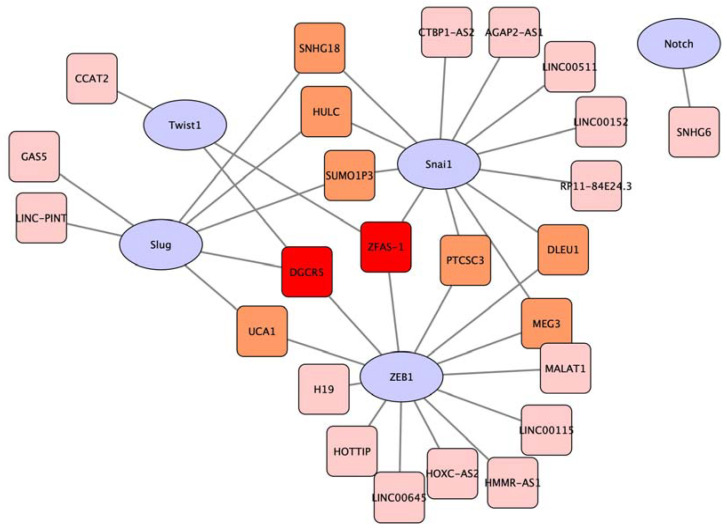
Network visualisation of various lncRNAs in regulating of EMT transcription factors. DGCR5 and ZFAS-1 (red box) were observed to be lncRNAs which were shown to be involved in the regulation of multiple EMT-TFs, namely Twist1, Slug, and ZEB1 (for DGCR5) and Twist1, Snai1, and ZEB1 (for ZFAS-1). Several lncRNAs (orange box) were identified to regulate two EMT-TFs, while lncRNAs in pink box were shown to only regulate a specific EMT-TF. Figure was created using Cytoscape software version 3.10.0.

Next, we identified lncRNAs that regulate transcriptional factors (TFs) of classical EMT biomarkers such as ZEB1, Twist, Notch, and Slug/Snai1 (Table 2). The knockdown of these lncRNAs (oncogenic) downregulated the expression of ZEB1, Twist, Notch, and Slug/Snai1 (Table 2) and vice versa in overexpression studies for the tumour-suppressive lncRNAs. From the studies, 13 lncRNAs were identified to regulate Snai1 and ZEB1, followed by Slug (6 lncRNAs), Twist1 (3 lncRNAs), and Notch (1 lncRNA) (Figure 2B). Some of the lncRNAs were reported to regulate multiple EMT-TFs (Figure 3), namely, (i) the downregulated lncRNA DGCR5 which downregulates TWIST1, Slug, and ZEB1 expression upon inactivation and (ii) upregulated lncRNA ZFAS-1 which upregulates TWIST1, ZEB1, and Snai1 expression upon overexpression. Only one lncRNA (SNHG6) was reported in the regulation of Notch.

### 3.4. Long Non-Coding RNAs Target EMT-Associated Biological Signalling Pathways

**Figure 4:**
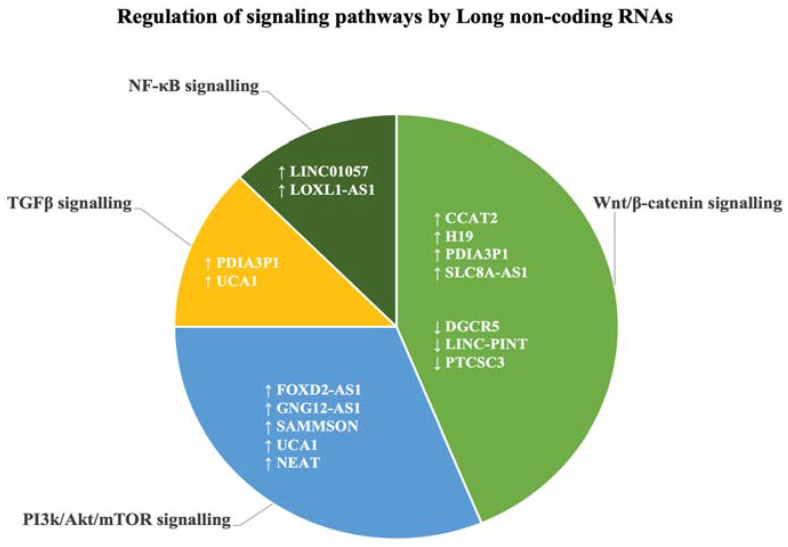
Upregulated (↑) and downregulated (↓) lncRNAs identified through systematic literature search which regulate biological signalling pathways prominent in GBM and cancers.

In our analysis, several studies reported that the GBM MES transition by lncRNAs involved specific signalling pathways, namely, Wnt/β-catenin, PI3K/Akt, TGF-β, NF-κB, and mTOR signalling pathways (Table 3). Amongst them, four upregulated (CCAT2, H19, PDIA3P1, SLC8A-AS1) and three downregulated (DGCR5, LINC-PINT, PTCSC3) lncRNAs were identified within Wnt/β-catenin signalling. We identified five upregulated lncRNAs (FOXD2-AS1, GNG12-AS1, SAMMSON, UCA1, and NEAT1) that were shown to regulate the PI3K/Akt/mTOR pathways, while the high expression of PDIA3P1 and UCA1 regulated the TGF-β pathway. Additionally, the upregulated lncRNAs LINC01057 and LOXL-AS1 were reported in the regulation of NF-κB signalling (Figure 4). Two lncRNAs were reported to be involved in regulating more than one signalling pathway, namely, UCA1 was reported to be involved in both PI3K/Akt and TGF-β signalling pathways, and PDIA3P1 was reported to be involved with both Wnt/β-catenin and TGF-β signalling pathways.

### 3.5. Factors Facilitating ECM Degradation Are Affected by lncRNAs’ Dysregulation

**Figure 5:**
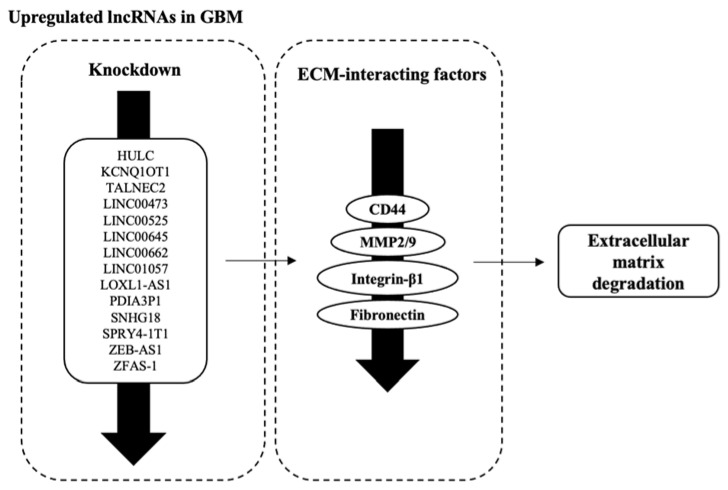
Downregulation of lncRNAs through knockdown led to the observation of decreased expression of factors integral to ECM integrity. The dysregulation of ECM factors and components will lead to the degradation of the ECM, which is part of the cytoskeletal remodelling process that promotes the invasive phenotype observed in GBM cells.

From the results, we obtained lncRNAs which interact with several factors that are integral in maintaining the integrity of ECM. These factors are CD44, MMP2/9, Integrin, and Fibronectin (Figure 5, Table 4). The downregulation of oncogenic lncRNAs through knockdown led to the subsequent downregulation of these ECM factors. Only one lncRNA, SLC8A1-AS1, was recorded to upregulate claudin expression following knockdown.

### 3.6. Downregulated lncRNAs and Their Potential Tumour-Suppressive Roles in GBM MES Transition

We identified 10 downregulated lncRNAs, which are tabulated in Table 5. As a contrast to the upregulated lncRNAs, the overexpression of the downregulated lncRNAs demonstrated the increased expression of E-cadherins and decreased expression of N-cadherin and vimentin. An opposite trend was observed where the inactivation of these downregulated lncRNAs is associated with decreased expression of E-cadherins and increased expression of N-cadherin and vimentin. Similarly, changes in the expression of these downregulated lncRNAs showed an opposite trend as well in the case of expression levels of the EMT-TFs (Slug, Snai1, ZEB1, and Twist) and ECM components (MMP2/9, fibronectin) which is inversely proportional to the expression of these downregulated lncRNAs.

### 3.7. Long Non-Coding RNAs Are Dysregulated in the Clinical Setting

Our analysis has observed a total of 41 lncRNAs from the 62 lncRNAs identified in our systematic search to be present in the clinical dataset (complete data available in File S1). Among these lncRNAs, 17 were found to be upregulated (FC > 2), while 8 were found to be downregulated (FC < 0.5). The top-10 upregulated and top-8 downregulated lncRNAs are tabulated in Table 6 and Table 7, respectively. Analysis was also performed to compare the expression of lncRNAs within GBM samples, including treatment received vs. no treatment received, primary vs. recurrent tumours, and IDH-wildtype versus IDH-mutant. No lncRNA of significance which is affiliated with these parameters was obtained.

### 3.8. Dysregulated lncRNAs Affect Cell Phenotype through Transcriptional and Translational Regulation

Next, we obtained the protein interactions of these lncRNAs from the RAID database (complete data available in File S1). We performed GSEA for the sets of proteins associated with upregulated lncRNAs (HOXAS3, H19, HOTTIP) and downregulated lncRNAs (MEG3, DGCR5, XIST) (data available in File S2. Figures S1–S6), GSEA through KEGG pathways demonstrated that the pathway “RNA transport” is enriched in three lncRNAs—HOXAS3, DGCR5, and XIST. GSEA through GO-BP and MF showed that the proteins associated with these lncRNAs mostly function as a transcriptional or translational control, with GO BP annotations related to translation, and transcription is highly enriched. GSEA through GO MF showed that these proteins primarily function through binding with RNA or binding at the transcription region.

## 4. Discussion

To date, the roles of any specific lncRNAs in cancers or specifically in GBM are still debatable. This is due to contradictory results in either pro- or anti-cancer effects or in terms of biological pathways that were observed between different cell lines and between cell and animal models. This was reflected similarly in our results, with the lncRNA MEG3 being an example. As the genetic makeup of cancer cells is highly heterogenous, it would be necessary to pinpoint the exact role of specific lncRNAs affected in any particular biological setting to allow the identification of the mechanism being regulated. Further development of lncRNAs without knowing their exact role within individualised cancer samples would be redundant.

### 4.1. EMT Markers E- and N-Cadherins Are Commonly Used as Markers of MES Transition in GBM

Both classical cadherins, E- and N-cadherins, were investigated in almost all of the studies as an indication of a GBM MES transition. As previously mentioned, cadherin expression in the neuronal setting has observed contradictory and inconsistent results. Noh et al. (2017) evaluated the expression of both cadherins in glioma tissues and cell lines and concluded their limited prognostic value in gliomas. With this in mind, developing a genomic profile for gliomas would provide a more accurate and indicative alternative to the current cadherin solution for the detection of an EMT-like process. Potential factors that would be a crucial element in such genomic profiles would be lncRNAs, EMT-TFs, and also activities of biological signalling pathways.

### 4.2. Transcription Factors for EMT Play an Important Role in the Overarching Regulation of MES Transition in GBM

Our analysis observed a huge number of lncRNAs that are capable of regulating the expression and activity of EMT transcription factors (ZEB1, Slug, Snail, Twist1) which modulate the expression of the typical EMT markers (E-cadherin, N-cadherin, and vimentin) and concomitantly induce MES transition in GBM. Among these transcription factors, Snai1 and ZEB1 are the most frequently studied and are regulated by 13 lncRNAs. These transcription factors are found to be the central regulators that modulate the expression of the EMT markers. For instance, Snai1 has been heavily implicated as the master regulator of EMT, where its overexpression can lead to the repression of E-cadherin [96]. Twist, consisting of Twist1/2, on the other hand, can also suppress E-cadherin which increases the expression of mesenchymal markers [97]. Another important transcription factor that drives GBM MES transition is ZEB1 [98].

In our analysis, we observed the interplays amongst the transcription factors, where ZEB1 activity was regulated by both Snai1 and Twist [99]. However, knowing the intrinsic mechanism among the transcription factors and whether their expressions are directly affected by lncRNA cannot be concluded at the current stage. Two lncRNAs identified in this study, DGCR5 and ZFAS-1, were shown to affect the expression of multiple EMT transcription factors. From our clinical database analysis, DGCR5 is one of the downregulated lncRNAs, which may suggest its tumour-suppressive role. To further verify the function of DGCR5, GSEA was performed on the on the proteins which were reported to interact with DGCR5. GSEA analysis inferred the potential translational silencing effect of DGCR5 on oncogenic target proteins via binding with a miRNA target in the canonical lncRNA–miRNA–mRNA signalling axis, which supports its tumour-suppressive role.

### 4.3. Transcription Factors of EMT Influence Components of Cancer-Associated Signalling Pathways Which Leads to GBM MES Transition

Our current analysis focuses the literature search on several prominent pathways which were reported to be affiliated with the EMT process. We observed several interplays among the signalling pathways that regulate the activity of the EMT transcription factors and EMT markers. PI3K signalling can be hyperactivated through activation of TGF-β, where TGF-β activation happens through the phosphorylation of Twist, leading to the subsequent observed loss of E-cadherin [100]. Downstream activation of PI3K leads to subsequent AKT and mTOR activation, which in turn upregulates Snai1 and also NF-κB. Meanwhile, the downstream activation of NF-κB activates multiple EMT transcription factors such as ZEB1, Slug, and Twist [101,102]. From our analysis, both PI3K and Wnt signalling are regulated by the highest numbers of lncRNAs. However, in these individual studies, the interplays between signalling pathways are often not investigated, which indicates the need for further validations on these lncRNAs to allow the identification of the specific molecular pathways affected. In hindsight, although our analysis demonstrated that lncRNAs regulate EMT transcription factors and EMT-associated biological signalling pathways, their direct association cannot be concluded at the current stage. For example, our systematic literature search demonstrated that the knockdown of H19 downregulated ZEB1 and Wnt signalling [60]. However, the direct correlation of Wnt signalling with ZEB1 regulation was unclear in the study. Moreover, the activity of Wnt can also be regulated through TGF-β, which is ZEB1-dependent. This further signifies the complex nature of lncRNA regulation of EMT at various biological levels.

### 4.4. Long Non-Coding RNAs Influence GBM Microenvironment through Various ECM Components Which Contribute to MES Transition

**Figure 6:**
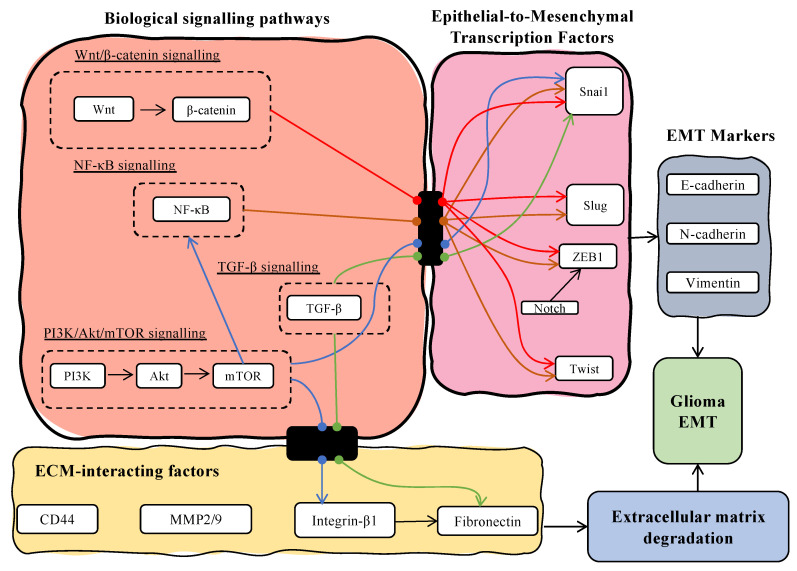
Pathway model in which various biological signalling pathways, including Wnt/β-catenin (red arrow), PI3K/Akt (blue arrow), TGF-β (green arrow), and NF-κB (brown arrow) signalling pathways will affect EMT-TFs and ECM-interacting factors which will subsequently induce the MES transition in GBM that is positively correlated with the expression of EMT markers, N-cadherin, and vimentin and negatively correlated with the expression of E-cadherin. The overarching regulation of lncRNA(s) was observed at all levels, which further emphasised the importance of accurate characterisation of the lncRNAs in future studies to allow the development of a promising lncRNA target(s) as either a biomarker or therapeutic target.

From our analysis, we also observed lncRNAs which modulated the expression of the ECM components, including matrix metalloproteins (MMPs), fibronectins, CD44, claudin, and integrin-β1. Matrix metalloproteinases (MMPs) are a family of endoproteases, which function to breakdown various proteins in the extracellular matrix (ECM), ultimately leading to their degradation [103]. Degradation of the ECM is closely associated with EMT and tumour cell invasion. In this sense, MMPs are also referred to as an initiator for EMT. In a clinical study, a low level of MMP2 is associated with longer mean survival amongst GBM patients, and its increased level is positively associated with increased tumour grades and recurrence [104]. In our analysis, the expression of two MMPs, MMP2 and MMP9, was frequently influenced by lncRNAs such as HULC, SNHG11, ZEB-AS1, and ZFAS-1. Additionally, both MMPs have been reported to be involved in ZEB1 signalling and Snai1 [105,106]. Similarly, there were lncRNAs identified (HULC, SNHG18, and ZFAS-1) which influence the expression of MMPs and are reported to regulate the EMT transcription factors. Based on this, a linkage between the lncRNAs that regulate the activity of the transcription factors (Snail and ZEB1), which in turn regulate the ECM components (including MMPs), can be observed during MES transition in GBM. Figure 6 illustrates the tight regulations of EMT-TFs and ECM-interacting factors by the signalling pathways highlighted in this study pertaining to the MES process in GBM. LncRNAs were reported to exhibit regulatory activity at various levels in this overarching signalling axis, which emphasised the importance of the accurate characterisation of lncRNAs’ roles within the GBM setting.

### 4.5. Research Is Lacking Outside of Cell Studies to Observe Clinical Relevance of the lncRNAs in GBM MES Transition

To obtain further insights into lncRNAs within the clinical setting, our analysis observed lncRNAs such as HOXC13-AS being similarly overexpressed in vitro and up to 68-fold in GBM tissues. Similarly, the downregulated expression patterns of MEG3, DGCR5, and LINC-PINT recorded from our literature search also match with the expression data in our analysis of GBM clinical samples (0.11, 0.12, and 0.50-fold, respectively). Interestingly, several upregulated lncRNAs obtained from our systematic search were found to be downregulated instead in the clinical setting. Examples of such lncRNAs are NEAT1 and UCA1, where both of which are widely studied oncogenic lncRNAs. A possible explanation for these inconsistencies could be attributed to the limitations of the current GBM experimental setup. Furthermore, additional analysis was performed to compare the expression of lncRNAs obtained in this study among various GBM clinical sample categories, including parameters such as intervention received and primary or recurrent tumours. From the analysis, only one lncRNA, LINC00645, was upregulated in temozolomide-treated GBM samples compared to non-treated GBM samples. This suggest that the exposure to TMZ may induce molecular interactions with the lncRNAs. Additionally, the exposure of TMZ was known to induce epigenetic changes, which may further affect or influence the expression of the lncRNAs. As for other lncRNAs subjected to TMZ-related analysis, no results of significance were obtained. This can be due to the following: (i) the expression of lncRNAs might not be affected by these parameters, (ii) the sample sizes of GBM clinical samples with these specific criteria are limited, and (iii) the out-of-date statuses of these publicly available expression profiles of GBM clinical samples have greatly limited the information available on the lncRNAs to be effectively compared.

Perhaps the most exigent need for EMT studies is to understand the in-depth process attributed to the high cell invasive phenotype of GBM, and this would expedite the development of anti-EMT therapies to palliate the current negative outcomes among GBM patients. Further, the EMT-like process and the associated factors discussed previously are also heavily implicated with the acquired chemoresistance, especially temozolomide (TMZ) resistance, in GBM. Although whether the acquisition of TMZ resistance and EMT are linked events remains unclear in our current understanding, some studies have reported the involvement of EMT factors in TMZ resistance, which include ZEB1, Slug, and vimentin [107,108,109].

### 4.6. Future Perspective in lncRNA Studies: Moving forward from Cell Studies

Practical application of lncRNAs in the GBM therapeutic setting is currently still at a mature stage. There are several early clinical trials which were carried out in other cancers but not in GBM [110]. Considering the heterogeneity observed within the genetic profiles between GBM patients, alongside the current practice for GBM diagnosis, development of an effective approach for GBM diagnosis is imperative. In this sense, lncRNAs serve as a potential tool that could be further developed for GBM diagnosis. However, much more effort is still required in characterising novel lncRNAs to allow a more in-depth understanding of the common genetic representation in GBM to allow the effective development of lncRNAs as reliable GBM genetic biomarkers. Additionally, it was seen that most research tends to focus on the upregulated, oncogenic lncRNAs compared to the tumour-suppressive lncRNAs. Future research can focus on tumour-suppressive lncRNAs as they can also be a potential molecular target in the EMT-like process in GBM. Additionally, the different expression profiles of some lncRNAs within in vitro models compared with those in a clinical setting accentuate the need for future studies using a GBM model with a closer-to-truth representation of genomic studies within the brain setting. There are several considerations that in vitro models will not be able to address fully, as they do not emulate the actual brain environment which will deaden the veracity of such studies. In contrast, 3D culture models could be a potential bridging tool to facilitate the convergence of 2D cell models to animal models. A systematic review performed by Phon et al. has found dissimilarities between genomic profiles of 3D and 2D culture models, where 3D cultures can simulate the brain microenvironment to a closer degree [111]. This further reinforces the need to verge upon the junctions between various pre-clinical models to allow the excogitation of lncRNA functions to a more precise level.

## 5. Conclusions

Our systematic analysis has identified upregulated and downregulated lncRNAs that confer oncogenic or tumour-suppressive properties that influence the GBM MES transition in GBM with a complex signalling regulation between the various EMT transcription factors, ECM components, and classical EMT markers (vimentin, E-cadherin, and N-cadherin). This GBM MES transition by lncRNAs is regulated by complex interplays between Wnt/β-catenin, PI3K/Akt, TGF-β, and NF-κB pathways. Nevertheless, the current understanding of the interplay between the signalling pathways and EMT factors is still immature. The complexity of various interplays between these factors implicated the importance of further validation studies to confirm the direct relation and crosstalk amongst factors in the signalling involved. Additionally, the disparity of lncRNAs’ expression further warrants the usage of more suitable pre-clinical models to allow the elucidation of these regulatory mechanisms relevant to the clinical settings.

## Figures and Tables

**Table 1:** Systematic literature search of upregulated lncRNAs that are involved in the regulation of GBM MES transition in GBM observed through the change in expression of EMT markers. The expression of these upregulated lncRNAs was inversely proportional to the expression of N-cadherin and vimentin, where an opposite trend in expression was observed in the case of E-cadherin.

lncRNA	miRNA Interactions	Knockdown or Overexpression Studies	Effect on EMT Markers Expression	References
AB073614	NA	Knockdown	↑ E-cadherin ↓ Vimentin	[21]
AGAP2-AS1	mi-497-5p	Knockdown	↑ E-cadherin ↓ N-cadherin	[22]
ASB16-AS1	NA	Knockdown	↑ E-cadherin ↓ Vimentin, N-cadherin	[23]
BLACAT1	NA	Knockdown	↑ E-cadherin ↓ Vimentin//N-cadherin	[24]
CCAT1	miR-181b	Knockdown	↑ E-cadherin ↓ Vimentin	[25]
CCAT2	NA	Knockdown	↑ E-cadherin ↓ Vimentin//N-cadherin	[26]
CTBP1-AS2	miR-370-3p	Knockdown	↑ E-cadherin ↓ Vimentin//N-cadherin	[27]
DANCR	miR-33a-5p	Knockdown	↑ E-cadherin ↓ Vimentin	[28]
DLEU1	NA	Knockdown	↓ N-cadherin	[29]
ENST01108	miR-489	Overexpression	↑ Vimentin ↓ E-cadherin	[30]
FOXD2-AS1	miR-506-5p, miR-185	Knockdown	↑ E-cadherin ↓ Vimentin//N-cadherin	[31,32,33]
GNG12-AS1	NA	Knockdown	↓ Vimentin	[34]
HMMR-AS1	NA	Both	Knockdown↓ Vimentin	[35]
HOTTIP	miR-101	Knockdown	↑ Vimentin ↓ E-cadherin	[36]
HOXA-AS3	miR-455-5p	Knockdown	↑ E-cadherin ↓ Vimentin//N-cadherin	[37]
HOXC13-AS	miR-122-5p	Knockdown	↓ Vimentin//N-cadherin	[38]
HSP90AA1-IT1	miR-885-5p	Knockdown	↓ Vimentin//N-cadherin	[39]
HULC	NA	Both	Knockdown ↑ E-cadherin ↓ Vimentin//N-cadherin Overexpression ↑ N-cadherin//Vimentin ↓ E-cadherin	[40]
KCNQ1OT1	miR-375	Knockdown	↑ E-cadherin ↓ Vimentin//N-cadherin	[41]
LBX2-AS1	miR-491-5p	Knockdown	↑ E-cadherin ↓ Vimentin//N-cadherin	[42]
LINC00115	miR-200s	Knockdown	↑ E-cadherin ↓ Vimentin	[43]
LINC00152	miR-612 and miR-107	Both	Overexpression ↑ N-cadherin//Vimentin ↓ E-cadherin	[44,45,46]
LINC00466	miR-598	Knockdown	↓ Vimentin//N-cadherin	[47]
LINC00473	miR-637	Knockdown	↑ E-cadherin ↓ N-cadherin	[48]
LINC00511	miR-524-5p miR-15a-5p	Knockdown	↑ E-cadherin ↓ Vimentin//N-cadherin	[49,50]
LINC00525	miR-338-3p	Knockdown	↑ E-cadherin ↓ Vimentin//Fibronectin	[51]
LINC00645	miR-205-3p	Both	Overexpression ↑ Vimentin	[52]
LINC00662	miR-34a-5p	Knockdown	↓ Vimentin	[53]
LINC01057	NA	Knockdown	↓ Vimentin	[54]
NEAT1	Mir-370-3p, miR-185-5p	Both	Knockdown ↑ E-cadherin ↓ Vimentin	[55,56]
NNT-AS1	miR-582-5p	Knockdown	↑ E-cadherin ↓ N-cadherin	[57]
PDIA3P1	miR-124-3p	Both	Knockdown ↓ N-cadherin//Vimentin Overexpression ↑ Vimentin	[58]
PVT1	miR-1207-3p	Knockdown	↓ N-cadherin//Vimentin	[59]
RP11-84E24.3	NA	Both	Overexpression ↑ N-cadherin//Vimentin ↓ E-cadherin	[60]
SAMMSON	NA	Knockdown	↑ E-cadherin ↓ N-cadherin	[61]
SLC8A1-AS1	NA	Knockdown	↑ E-cadherin ↓ N-cadherin//Vimentin	[62]
SNHG11	miR-154-5p	Knockdown	↓ N-cadherin//Vimentin	[63]
SNHG18	NA	Knockdown	↑ N-cadherin//Vimentin ↓ E-cadherin	[64]
SNHG6	miR-101-3p	Knockdown	↑ E-cadherin ↓ Vimentin	[65,66]
SPRY4-IT1	NA	Knockdown	↑ E-cadherin ↓ Vimentin	[67]
SUMO1P3	NA	Both	Knockdown ↑ E-cadherin ↓ N-cadherin//Vimentin	[68]
UCA1	miR-1, miR-203a, miR-204-5p, miR-135a, miR-206	Both	Knockdown ↑ E-cadherin ↓ N-cadherin//Vimentin Overexpression ↑ N-cadherin//Vimentin ↓ E-cadherin	[69,70,71,72,73]
XIST	miR-133a	Knockdown	↑ E-cadherin ↓ N-cadherin//Vimentin	[74]
ZEB1-AS1	NA	Knockdown	↑ E-cadherin ↓ N-cadherin	[75]
ZFAS-1	NA	Knockdown	↑ E-cadherin ↓ N-cadherin	[76,77]

lncRNA: long non-coding RNA; miRNA: microRNA. **↓**: downregulated; **↑**: upregulated.

**Table 2:** Systematic search of the upregulated lncRNAs in regulating EMT transcription factors associated with GBM MES transition in GBM. The expression of these upregulated lncRNAs was observed to be directly proportional to the expression of the EMT-TFs listed.

lncRNA	miRNA Interactions	Knockdown or overexpression Studies	Effect of lncRNA Expression on EMT-TFs	References
AGAP2-AS1	mi-497-5p	Knockdown	↓ Snai1	[22]
CCAT2	NA	Knockdown	↓ Twist//Snail//β-catenin	[26]
CTBP1-AS2	miR-370-3p	Knockdown	↓ Snai1	[27]
DLEU1	NA	Knockdown	↓ ZEB1//Snai1	[29]
H19	miR-130a-3p, miR-200a	Both	Overexpression: ↑ ZEB1	[78,79,80]
HMMR-AS1	NA	Both	Knockdown: ↓ ZEB1	[35]
HOTTIP	miR-101	Knockdown	HOTTIP indirectly upregulates ZEB1	[36]
HOXC-AS2	miR-876-5p	Knockdown	↑ ZEB1, lncRNA also positively regulated by ZEB1 feedback loop	[81]
HULC	NA	Both	Overexpression: ↑ Slug//Snai1	[40]
LINC00115	miR-200s	Knockdown	↓ ZEB1	[43]
LINC00152	miR-612 and miR-107	Both	Overexpression: ↑ Snai1	[44,45,46]
LINC00511	miR-524-5p miR-15a-5p	Knockdown	↓ Snai1	[49,50]
LINC00645	miR-205-3p	Both	Knockdown: ↓ ZEB1	[52]
MALAT1	NA	Both	Overexpression: ↑ ZEB1	[82]
RP11-84E24.3	NA	Both	Knockdown: ↓ Snai1	[60]
SNHG18	NA	Overexpression	↑ Snai1//Slug	[64]
SNHG6	miR-101-3p	Knockdown	↓ Notch1	[65,66]
SUMO1P3	NA	Both	Knockdown: ↓ Slug//Snai1	[68]
UCA1	miR-1, miR-203a, miR-204-5p, miR-135a, miR-206	Both	Knockdown: ↓ Slug1//ZEB1	[69,70,71,72,73]
ZFAS-1	NA	Knockdown	↓ Snai1//ZEB1//Twist	[76,77]

lncRNA: long non-coding RNA; miRNA: microRNA. **↓**: downregulated; **↑**: upregulated.

**Table 3:** List of upregulated lncRNAs which regulate EMT-associated signalling pathways.

lncRNA	Knockdown or Overexpression Studies	Effect of lncRNA Expression	References
CCAT2	Knockdown	↓ β-catenin	[25]
FOXD2-AS1	Knockdown	↓ AKT1	[31,32,33]
GNG12-AS1	Knockdown	↓ P-AKT	[34]
H19	Both	LncRNA affects Wnt/β-catenin pathway	[78,79,80]
LINC01057	Both	LncRNA activates NF-κB	[54]
LOXL1-AS1	Knockdown	LncRNA activates NF-κB indirectly through RelB repression	[83]
NEAT1	Both	Overexpression: ↓ DNMT1 DNMT1 affects mTOR signalling	[55,56]
PDIA3P1	Both	Knockdown: ↓ β-catenin//TGF-β Overexpression: ↑ TGF-β RELA expression directly proportional to NF-κB activity	[58]
SAMMSON	Knockdown	Knockdown inactivated PI3K/Akt pathway	[61]
SLC8A1-AS1	Knockdown	SLC8A1-AS1 knockdown impairs Wnt/β-catenin signalling	[62]
UCA1	Both	Knockdown: ↓ p-AKT LncRNA involves in TGF-β signalling	[69,70,71,72,73]

lncRNA: long non-coding RNA; miRNA: microRNA. **↓**: downregulated; **↑**: upregulated.

**Table 4:** Upregulated lncRNAs which regulate factors and components that affect the integrity of the extracellular matrix.

lncRNA	Knockdown or Overexpression Studies	Effect of lncRNA Expression	References
HULC	Both	Overexpression: ↑ MMP2//MMP9	[40]
KCNQ1OT1	Knockdown	↓ MMP9	[41]
TALNEC2	Knockdown	↓ Fibronectin	[44]
LINC00473	Knockdown	↓ MMP9//CDK6	[48]
LINC00525	Knockdown	↓ Fibronectin	[51]
LINC00645	Both	Overexpression: ↑ Fibronectin	[52]
LINC00662	Knockdown	↓ Fibronectin	[53]
LINC01057	Both	↓ CD44	[54]
LOXL1-AS1	Knockdown	↓ CD44	[83]
PDIA3P1	Both	Knockdown: ↓ CD44	[58]
SLC8A1-AS1	Knockdown	↑ Claudin	[62]
SNHG18	Overexpression	↑ MMP2//MMP9	[64]
SPRY4-IT1	Knockdown	↓ Fibronectin	[67]
ZEB1-AS1	Knockdown	↓ MMP2//MMP9//Integrin-β1	[75]
ZFAS-1	Knockdown	↓ MMP2//MMP9//Integrin-β1	[76,77]

lncRNA: long non-coding RNA; MMP: Matrix Metalloprotein. **↓**: downregulated; **↑**: upregulated.

**Table 5:** Downregulated lncRNAs which act with a tumour-suppressive role in the GBM MES transition in GBM.

lncRNA	miRNA Interactor	Knockdown or Overexpression Studies	EMT Suppressed/Activated	References
CASC2	miR-18a	Knockdown	↑ Vimentin//N-cadherin ↓ E-cadherin	[84]
DGCR5	miR-21 and miR-23a	Overexpression	↑ E-cadherin//ZO-1//β-catenin ↓ Vimentin//Snai2//Twist//ZEB1//Fibronectin	[85,86]
GAS5	miR-106b	Overexpression	↑ E-cadherin ↓ Slug//Vimentin GAS5 regulates PTEN through miR-106b	[87]
LINC-PINT	NA	Overexpression	↓ N-cadherin//Vimentin//Slug LINC-PINT also suppresses Wnt/β-catenin signalling	[88]
LINC00312	miR-21-3p	Overexpression	↑ E-cadherin ↓ Vimentin//N-cadherin//MMP2//MMP9	[89]
LINC00599	NA	Overexpression	↑ E-cadherin ↓ Vimentin	[90]
LINC00961	NA	Overexpression	↑ E-cadherin ↓ Vimentin//N-cadherin	[91]
MEG3	miR-6088	Both	Knockdown: ↑ E-cadherin ↓ Vimentin//N-cadherin//Snai1 Overexpression: ↑ ZEB1//ZEB2 Regulates SMARCB1 through miR-6088 Also reported to have both oncogenic and tumour-suppressive effects in two separate studies	[92,93]
PTCSC3	NA	Overexpression	↑ E-cadherin ↓ Fibronectin//Snai1//ZEB1 Overexpression also inhibits Wnt/β-catenin pathway	[94]
TCONS_00020456	NA	Knockdown	↑ N-cadherin//Vimentin ↓ E-cadherin	[95]

lncRNA: long non-coding RNA; miRNA: microRNA. **↓**: downregulated; **↑**: upregulated.

**Table 6:** Top-10 upregulated lncRNAs in GBM clinical setting.

lncRNA	Fold Change
HOXC13-AS	68.26
HOXA-AS3	50.15
H19	31.32
HOXC-AS2	26.98
HOTTIP	11.36
SLC8A1-AS1	9.88
AGAP2-AS1	9.49
LBX2-AS1	6.07
LINC00511	5.44
DLEU1	4.57

**Table 7:** Top-8 downregulated lncRNAs in GBM clinical setting.

lncRNA	Fold Change
MEG3	0.11
DGCR5	0.12
MALAT1	0.30
XIST	0.33
BLACAT1	0.34
KCNQ1OT1	0.36
SAMMSON	0.46
LINC-PINT	0.50

## Data Availability

Not applicable.

## Supplementary Materials

The following supporting information can be downloaded at: https://www.mdpi.com/article/10.3390/biology12060818/s1, File S1: List of dysregulated lncRNAs identified in clinical dataset and all lncRNA-protein interactions obtained from RAID database for selected lncRNAs; File S2: GSEA of lncRNAs (HOXAS3, H19, HOTTIP, MEG3, DGCR5, and XIST) through GO-BP, GO-MF, and KEGG Pathways.
