# Supplementary material for: Regulation of EMT Markers, Extracellular Matrix, and Associated Signalling Pathways by Long Non-Coding RNAs in Glioblastoma Mesenchymal Transition: A Scoping Review
Source: Biology (Basel). 2023 Jun 4;12(6):818. doi: 10.3390/biology12060818 (PMC10294841; doi:10.3390/biology12060818)
Supplement: Supplementary file 1 [file biology-12-00818-s001.zip › File S2.pdf]

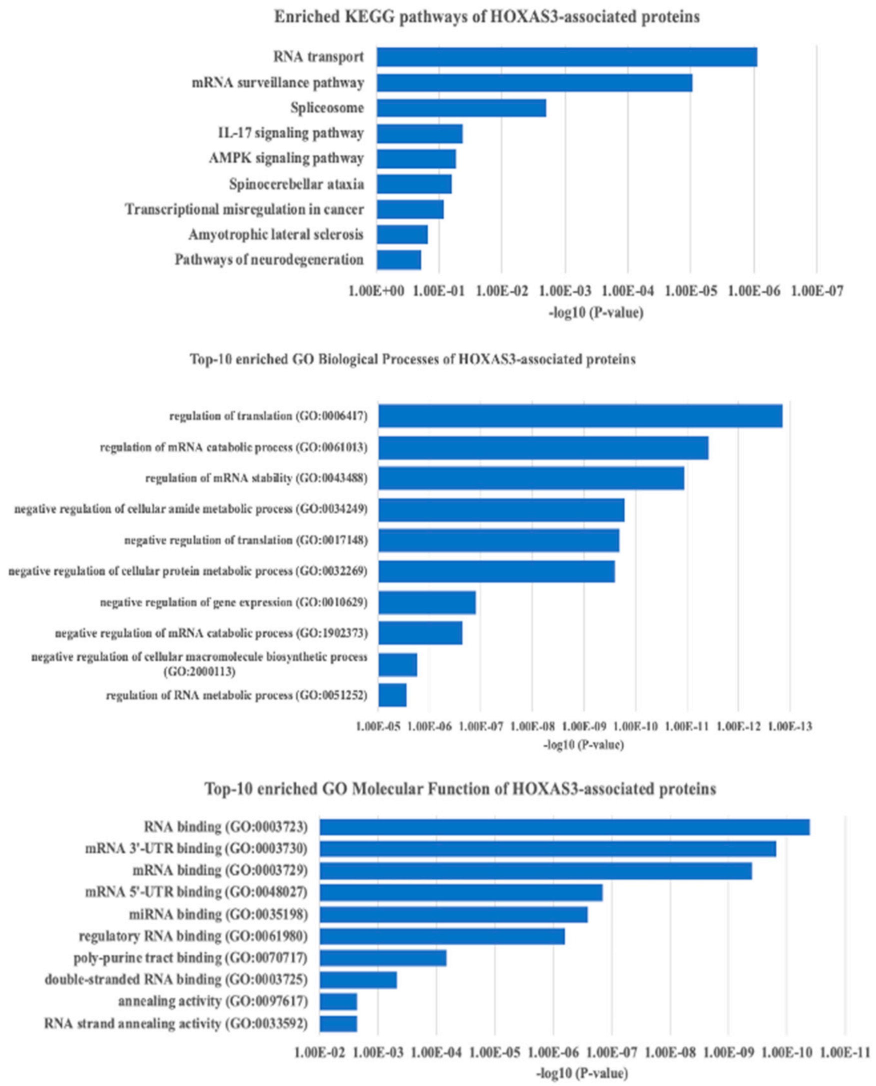

Figure S1: Gene set enrichment analysis of HOXAS-3-associated proteins.

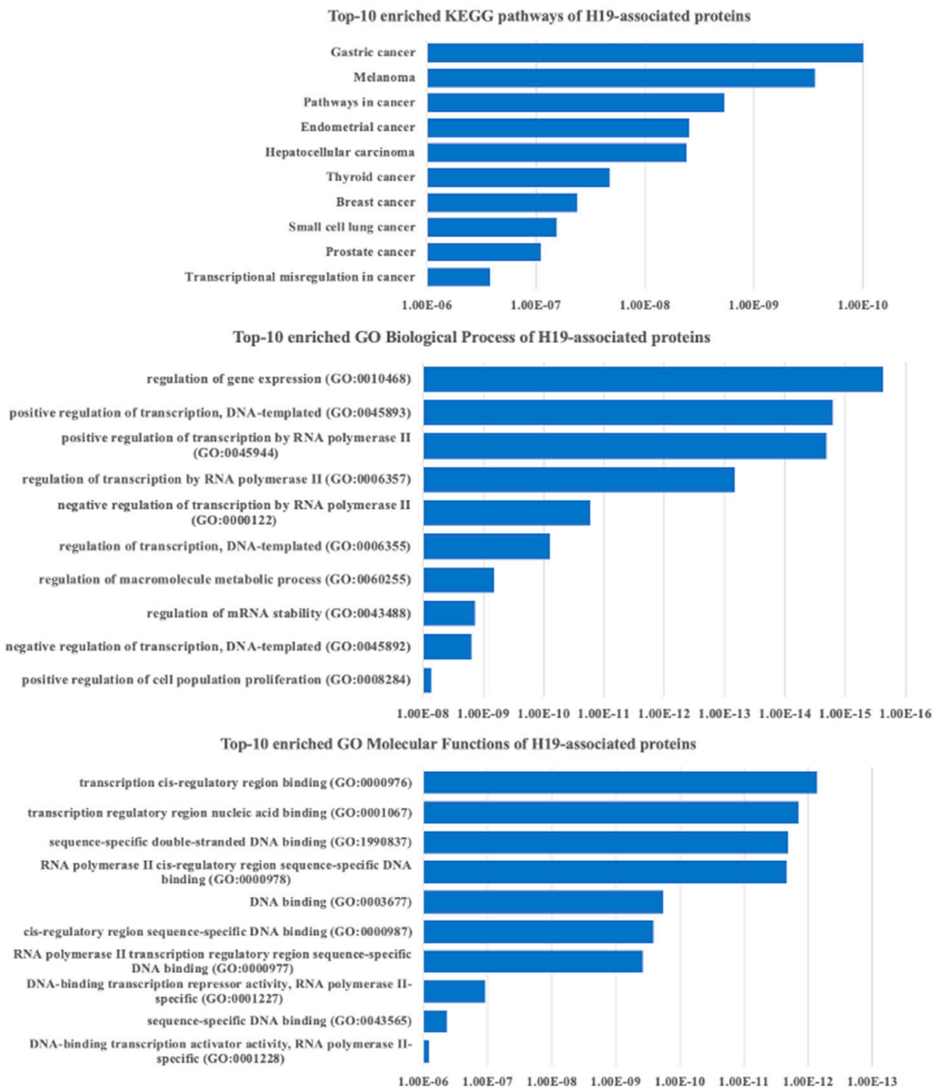

Figure S2: Gene set enrichment analysis of H19-associated proteins.

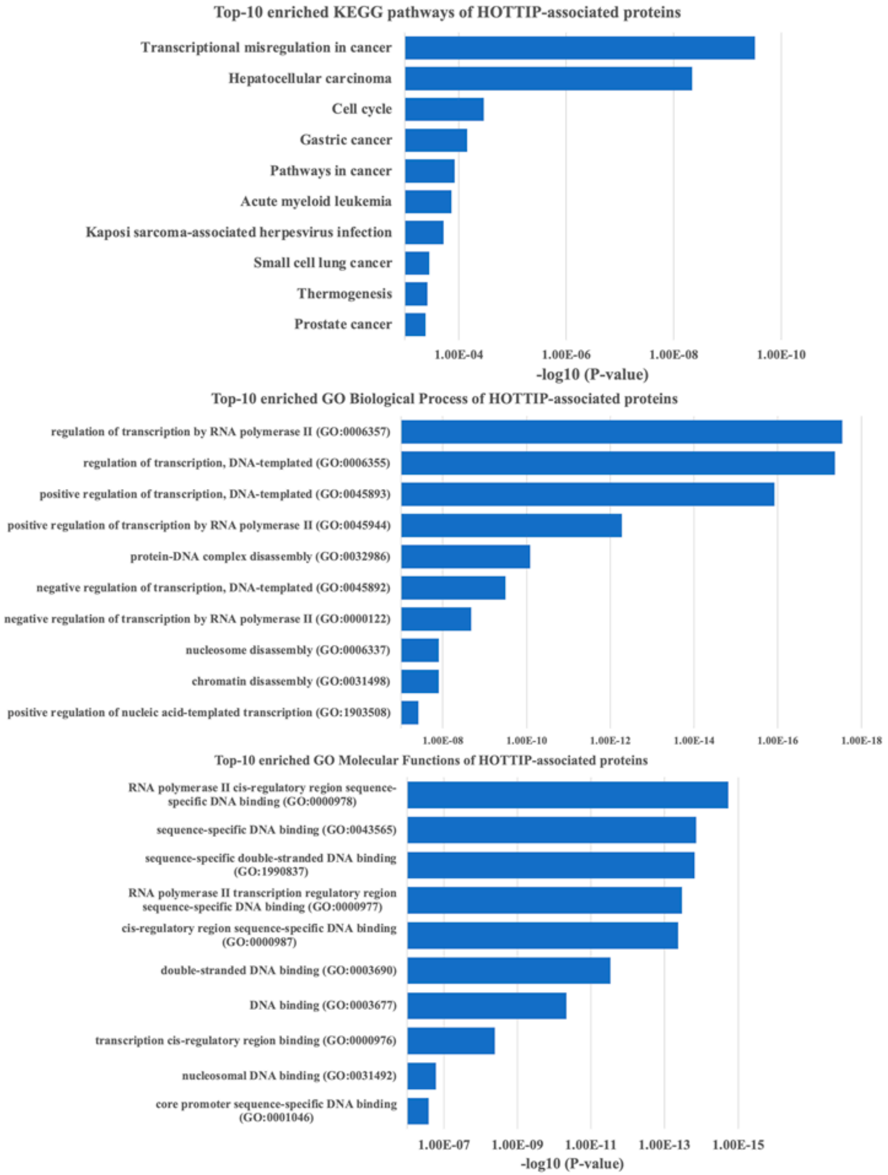

Figure S3: Gene set enrichment analysis of HOTTIP-associated proteins.

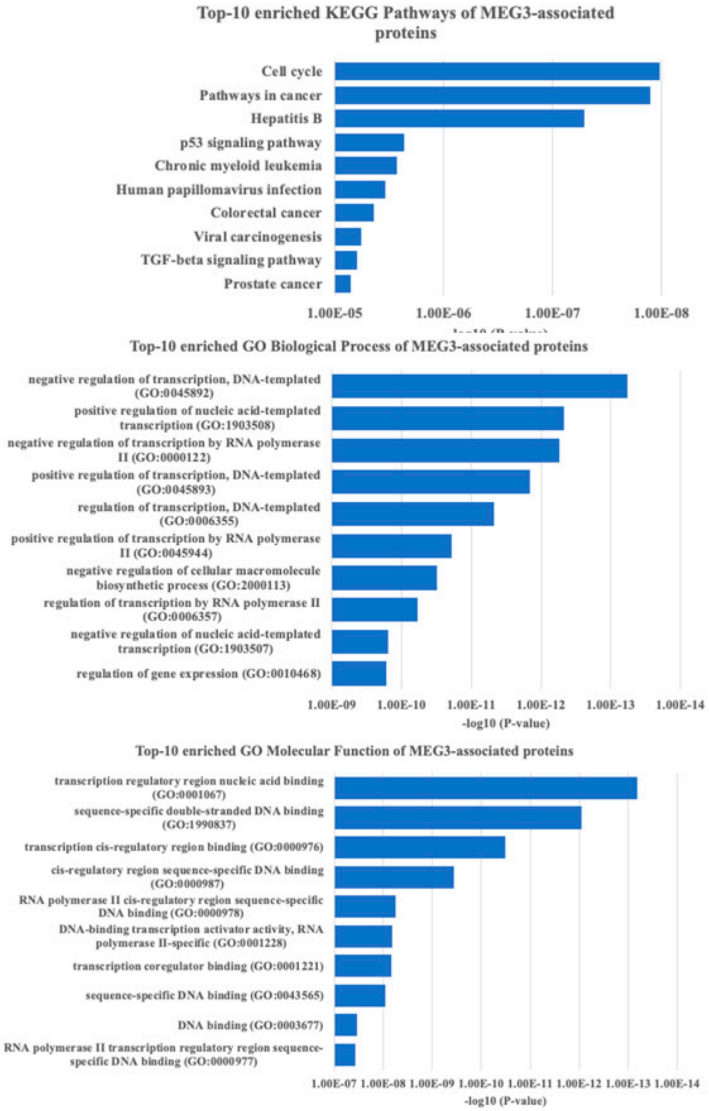

Figure S4: Gene set enrichment analysis of MEG3-associated proteins.

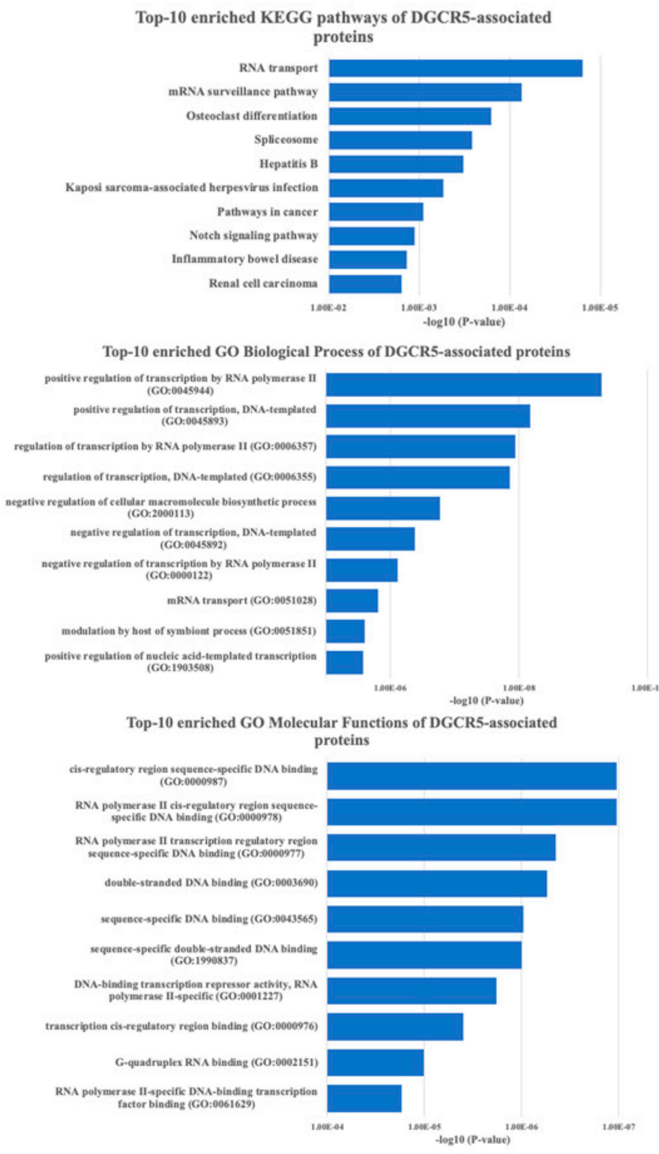

Figure S5: Gene set enrichment analysis of DGCR5-associated protein.

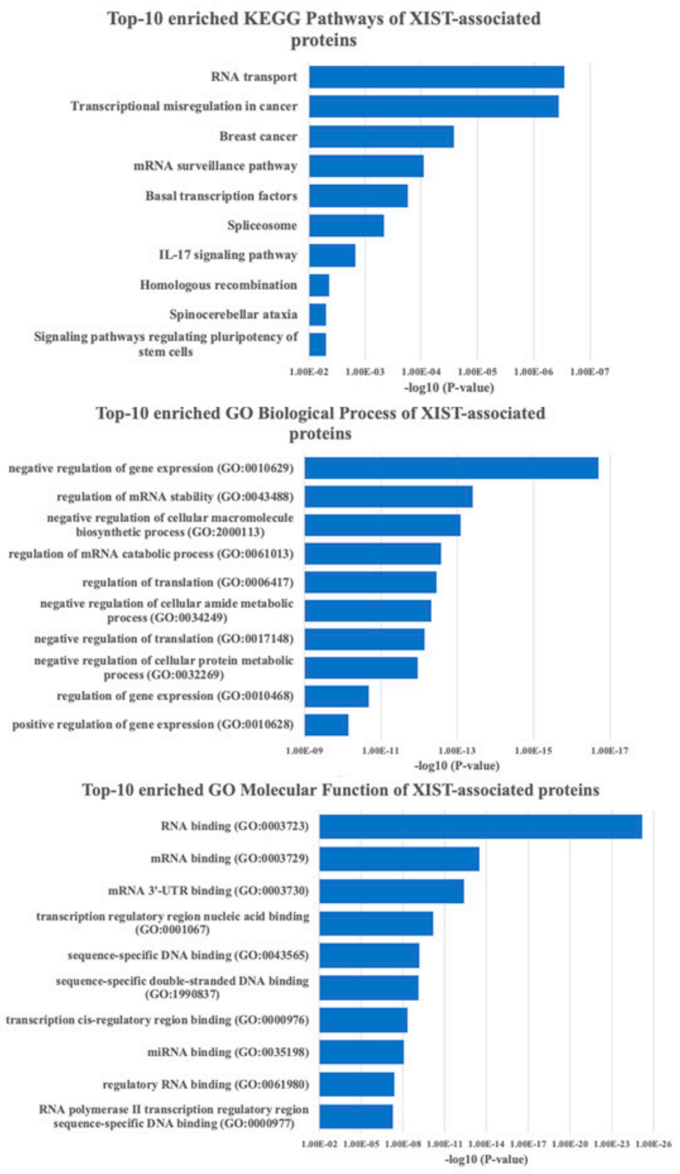

Figure S6: Gene set enrichment analysis of XIST-associated proteins.
